# Toward a Unified Socio-Cognitive Framework for Salience in Language

**DOI:** 10.3389/fpsyg.2016.01110

**Published:** 2016-08-05

**Authors:** Hans-Jörg Schmid, Franziska Günther

**Affiliations:** Department of English and American Studies, Ludwig Maximilian University of MunichMunich, Germany

**Keywords:** salience, expectation, contexts, language experience, social group

## Introduction: opposing views of salience

To begin with, consider the following four statements, one by one:

1. The word seemed salient because it was the first word that came to my mind.
2. The word seemed salient because it was the first word that came to my mind in this context.
3. The word seemed salient because I had not expected to hear it in this context.
4. The word seemed salient because I had never heard it before.

It is not unlikely that all four statements seem plausible, although 1 and 2 are actually opposed to 4 and 3 respectively. Apparently, then, words can be considered salient because they are…

1. highly familiar and strongly entrenched,
2. highly expected in a given context,
3. highly unexpected in a given context, or
4. totally unfamiliar.

Surprisingly, linguists have actually relied on at least three of these four scenarios for defining the notion of salience (see also Bowman et al., 2013, for a psychological perspective). Scenario (1) lies at the heart of Giora's idea of salience as what is “foremost on one's mind […] stored and coded in the mental lexicon” (Giora, 2003, p. 15). Scenario (2) accords with Geeraerts' view of onomasiological salience in terms of “the relative frequency with which a signifiant is associated with a given signifié” (Geeraerts, forthcoming), i.e., the frequency with which a word is used to denote a given piece of experience. Scenario (3) corresponds to understanding salience in terms of surprisal, as, e.g., proposed by Rácz: “A segment is cognitively salient if it has a large surprisal value when compared to an array of language input” (Rácz, 2013, p. 37; see also Friston, 2010; Clark, 2013; Fine et al., 2013; Divjak, 2016). Scenario (4) represents an extreme variant of type (3) which builds on memory-based *novelty* rather than context-based *surprise* (see Barto et al., 2013, for this distinction).

The four scenarios can be summarized systematically by a cross-tabulation of two types of sources of expectations, viz. long-term memory and current context, with two types of mechanisms of salience, viz. confirmation and violation of expectations:

1. Salience by context-free entrenchment: confirmation of expectations based on knowledge stored in long-term memory.
2. Salience by contexual entrenchment: confirmation of expectations derived from the probability of occurrence in the current context.
3. Salience by surprisal: violation of expectations derived from the probability of occurrence in the current context.
4. Salience by novelty: violation of expectations based on lack of stored knowledge.

In this paper we propose a unified framework for salience which reconciles these opposing conceptions by showing that they focus on different aspects of the interaction between knowledge, context, expectation, and external input.

## Expectation and types of contexts

Recent theories of linguistic and general perceptual, cognitive, and/or neural systems and processing share the view that expectations primed by context are crucial for salience effects to occur (see Levy, 2008; Friston, 2010; Clark, 2013; Fine et al., 2013; Jaeger and Snider, 2013; Divjak and Caldwell-Harris, 2015, pp. 59–60). The notions of *expectation* and *context* thus seem to hold the key to a better understanding of salience.

We define *expectation* as the state of the cognitive system immediately prior to processing a given linguistic cue. This state represents the *immediate cognitive context* for the upcoming processing event. What becomes activated as immediate cognitive context results from the interaction between four types of input which we also regard as contexts:

(1a) *Linguistic context*, i.e., what has been said before
(1b) *Situational context*, i.e., the participants, time, place, setting, objects
(1c) *Social context*, i.e., the type of social event, the social roles of and relations between participants
(2) *General cognitive context*, i.e., general and linguistic knowledge and routines stored in long-term memory.

**Figure 1:**
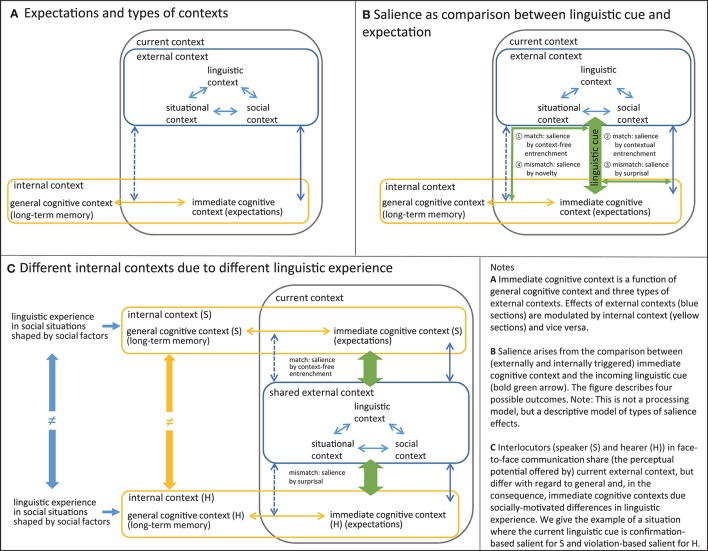
**Embedding salience effects in a generalized contextual interaction framework. (A)** Expectations and types of contexts. **(B)** Salience as comparison between linguistic cue and expectation. **(C)** Different internal contexts due to different linguistic experience.

All four types of contexts cooperate in shaping the immediate cognitive context, and yet type (2) differs fundamentally from the other three types. Whereas types (1a–c) are based on the current perception of external events, general cognitive context is internal and based on long-term memory. However, as has been acknowledged in the psychological literature on salience and attention (e.g., Wilder et al., 2011; Clark, 2013), the effects of perception-based external contexts on our immediate cognitive contexts are invariably modulated by our memory-based general cognitive contexts, because what we perceive, how we perceive it, and how we process it linguistically is strongly affected by what we already know. In addition to this interaction between current external contexts and long-term internal context (see also Fine et al., 2013), the three types of external contexts—linguistic, situational, and social—also influence each other. For example, the perception of the linguistic context will partly depend on that of the situational and social context in the use of deictic expressions such as *the book over there* or the use of forms of address like *Madam* or *Doctor*. A graphic representation of this view of expectation and context is provided in Figure 1A.

## Salience as comparison between the incoming linguistic cue and immediate cognitive context

Salience effects arise when an incoming linguistic cue is processed before the backdrop of the immediate cognitive context. Since salience effects are considered to involve the confirmation or violation of expectations (see Introduction: Opposing Views of Salience), the notion of salience—both in perception and in language—logically depends on a comparison between expectations and the cue to be processed. This characteristic is shared by perceptual and linguistic salience. A word that is surprising in a given linguistic or situational context (see Scenario 3 in Introduction: Opposing Views of Salience) is salient by virtue of the same principle as a green apple is in an array of red apples, i.e., through a comparison of a piece of information against its context. What is special about salience in language is that linguistic context plays a key role, and that general long-term memory-based context includes the full range of entrenched linguistic knowledge and routines, i.e., the individual's current linguistic competence.

## Different views of salience highlight different outcomes of the comparison

We would like to argue that the seemingly opposing types of salience explained in the introduction correspond to four different outcomes of the comparison between the immediate cognitive context and its sources, and the incoming linguistic cue (see Figure 1B).

1. Salience by context-free entrenchment: the incoming cue matches expectations that are mainly activated from general cognitive context, i.e., linguistic knowledge stored in long-term memory; this is the case for a very frequent word that is generally highly familiar and strongly entrenched, irrespective of the current context.
2. Salience by contextual entrenchment: the incoming cue matches expectations whose activation is mainly triggered by current linguistic, situational, and/or social context; examples are words that are strongly suggested by what was said before (e.g., as part of a strong collocation), by situational circumstances (e.g., by reference to a salient object), or by social aspects of the speech event (e.g., in a ritualized speech event like a baptizing or wedding ceremony).
3. Salience by surprisal: the incoming cue fails to match expectations that are mainly activated from current linguistic, situational, and/or social context; this could arise from violations of collocational restrictions or preferences, from unfamiliar ways of referring to objects, or from different conceptions of the social significance of words.
4. Salience by novelty: the incoming cue completely fails to match up with expectations activated from long memory; the hearer encounters a word that he or she simply does not know.

The four different views of salience thus highlight different interactions between internal and external contexts as sources of salience on the one hand, and the mechanisms of confirmation and violation of expectations on the other. The main step forward made by the integrative and unified view we are proposing consists in the way in which it integrates internal and external as well as long-term and short-term contextual effects. This characteristic of the model opens up further options for explaining interactional and social salience effects that we have neglected so far because we have focused on an individual idealized speaker.

## Violation-based salience in interaction can arise from experience-based social differences between speakers

Linguistic salience effects arise in the interaction between two or more interlocutors. So the framework proposed thus far must be extended. Figure 1C represents the idealized case of two participants, a speaker (S) and hearer (H), engaged in face-to-face interaction. As is indicated in the Figure, in this case the participants largely share the same external linguistic, situational, and social context. The impact of these external contexts on their respective immediate cognitive contexts is not identical, however, partly because the participants may not have equal perceptual access to what was said before or to objects in the shared situation. More importantly, and as pointed out above, the effect of external context is modulated by internal long-term knowledge, which is by definition individual rather than shared (see Fine et al., 2013, p. 1), and therefore differs from speaker to speaker (as is indicated by the arrows interrupted by the “is unequal” symbol).

The effect of these differences is that, despite shared external context, the current expectations of the two participants differ because the linguistic and encyclopedic knowledge they recruit for shaping their immediate cognitive contexts is not the same. Figure 1C illustrates a case where a linguistic cue (e.g., a word) that is highly familiar to the speaker is contextually surprising to the hearer. Such a word would be confirmation-based salient for the speaker, but violation-based salient for the hearer if the latter does not expect the word in this context or has never heard it before.

The likelihood of such situations correlates with the difference between the participants' general cognitive contexts, i.e., their entrenched linguistic association patterns and routines. According to usage-based models of grammar (e.g., Barlow and Kemmer, 2000; The Five Graces Group, 2009) these patterns and routines are shaped by lifelong linguistic experience, which is in turn shaped by social factors such as group-membership and participation in social networks and communities of practice (Schmid, 2015; see the left-hand side of in Figure 1C). At this point, the cognitive dimension of the framework is supplemented by the social dimension. While the cognitive dimension highlights the existence of individual differences, the social dimension licenses testable predictions concerning the sources of these differences and their effects on salience. One such prediction is that interlocutors from distant social groups in terms of education, age, ethnicity, gender, and other classic social variables are more likely to experience violation-based salience effects—“I have never heard this before,” “I would not have expected this in this context”—than interlocutors who share their social background and linguistic experience. In this way, our framework naturally integrates salience effects typically observed in sociolinguistic conceptions of salience. We therefore regard is as an integrative and unifying socio-cognitive framework for understanding salience. The paper by Jaeger and Weatherholtz (2016) in this special issue, which accords extremely well with the ideas presented here, provides more details and empirical evidence concerning the sociolinguistic aspects.

## Conclusion

We have proposed a unified framework which reconciles the tension between opposing views of salience by means of a differentiated conception of two central elements of salience, viz. *expectation* and *context*. Linguistic salience emerges from a comparison between an incoming linguistic cue and expectations that are activated from the interaction between current perception-based linguistic, situational, and social context, and long-term memory-based cognitive context (i.e., linguistic and encyclopedic knowledge). Different existing conceptions of salience highlight different aspects of this coherent framework. Experientially and socially motivated differences between the long-term memory-based cognitive contexts of individuals can be responsible for surprisal-based salience effects. The framework proposed is thus *socio-cognitive* in the sense that it accommodates both cognitive and social causes of linguistic salience effects.

## Author contributions

All authors listed have made substantial, direct and intellectual contribution to the work, and approved it for publication.

### Conflict of interest statement

The authors declare that the research was conducted in the absence of any commercial or financial relationships that could be construed as a potential conflict of interest.
